# Evaluation of the effectiveness of the standard traditional Korean medicine-based health promotion program for disadvantaged children in South Korea

**DOI:** 10.1186/s12906-022-03634-w

**Published:** 2022-06-26

**Authors:** Eunhye Hyun, Jiseon Ryu, Kibong Kim, Sangjae Lee, Seungtae Kim, Byungmook Lim

**Affiliations:** 1 Division of Humanities and Social Medicine, School of Korean Medicine, Pusan National University, Yangsan, South Korea; 2 Department of Korean Medicine, Danasa Nursing Hospital, Busan, South Korea; 3 Department of Pediatrics, School of Korean Medicine, Pusan National University, Yangsan, South Korea; 4 Division of Longevity and Biofunctional Medicine, School of Korean Medicine, Pusan National University, Yangsan, South Korea; 5 Division of Meridian and Structural Medicine, School of Korean Medicine, Pusan National University, Yangsan, South Korea

**Keywords:** Traditional Korean Medicine, Disadvantaged children, Health promotion, Quasi-experimental design, Difference-in-differences model

## Abstract

**Background:**

Traditional Korean Medicine (TKM) is highly integrated with the modern health care system of South Korea and is actively used in the public health field. Since 2014, the Ministry of Health and Welfare of South Korea has supported the development of standard models for TKM-based health promotion programs. This study aimed to develop and evaluate a standard TKM-based health promotion program for disadvantaged children.

**Methods:**

Using convenience sampling, we recruited 16 Community Children’s Centers (CCCs) located in Busan and Yangsan, South Korea, which are welfare daytime facilities for children from socially disadvantaged families. The CCCs were divided into two groups of eight CCCs—intervention CCCs and control CCCs—through random allocation, and children in each group were selected as subjects for the study. For 12 weeks, the TKM-based health promotion program developed in this study along with the basic services of CCCs were applied to children in the intervention group, and only the basic services of CCCs were provided to children in the control group. Data were obtained through pre- and post-surveys with the legal representatives of the children prior to implementing the program and after the 12-week program, respectively. The outcome variables—the number of outpatient visits, absences, lateness/early leaves, infectious symptoms, and EuroQol-5D and EQ-visual analog scale scores–were measured and statistically compared between the groups by descriptive analysis, chi-square test, t-test, and difference-in-differences model with regression analysis.

**Results:**

At baseline, there were 156 children in the intervention group and 153 children in the control group, among which 155 and 147 children, respectively, were included in the analysis. Results indicated that the number of outpatient visits was significantly lower (by 65%) in the intervention group than in the control group (*p* = 0.03), and this was similar in the sensitivity analysis. Regarding other outcome variables, the effects were not consistently significant.

**Conclusions:**

A standard TKM-based health promotion program has the potential to improve the health of disadvantaged children. In the future, studies with long-term interventions and a larger sample are needed to enhance the applicability of these programs in communities.

**Supplementary Information:**

The online version contains supplementary material available at 10.1186/s12906-022-03634-w.

## Background

It is well known that individuals in unfavorable environments are more susceptible to negative health outcomes [1]. Specifically, a child raised in a structurally or economically disadvantaged parenting environment is likely to have lower socioeconomic status and poorer health conditions in adulthood [2–4]. Therefore, a proactive approach to children’s health issues is required at the social level [5].

In South Korea, both conventional and traditional Korean Medicine (TKM) are used to provide social support for children’s health. TKM has been highly integrated into the nation’s healthcare system, and many TKM procedures provided at hospitals or clinics have been covered by the National Health Insurance since 1987 [6]. The use of TKM in the public health field began during the early 2000s, when the Ministry of Health and Welfare encouraged community health centers to implement TKM-based health promotion programs reflecting the health-related needs of community residents [7]. Thus, various TKM-based health promotion programs were conducted, such as Qi-gong (氣功) exercise classes, stroke prevention classes, Sa-sang (四象) constitution health classes, pre- and post-natal health classes, child-caring classes, smoking cessation classes, and home visit care services [7, 8]. Such programs increased community residents’ interest in health promotion and yielded high satisfaction and positive outcomes [7–11].

However, as the programs were autonomously introduced at each community health center, several problems emerged. Specifically, differences in budget levels and human resources in each community resulted in the relevant departments or personnel either being excessively deployed or insufficient [12, 13]. In particular, the lack of resources caused the quality of the projects to stagnate, which subsequently reduced participation by community residents. This hindered the recruitment of participants for evaluating the program’s performance, thereby limiting any program evaluation [7, 12]. In addition, over half of the public health TKM doctors employed at community health centers at the time complained that there was no systematic manual, not only for the application of the program, but also for evaluating its performance. This led to poor participation of key staff [14].

To address these problems, the Ministry of Health and Welfare, with its affiliate organization, the Korea Health Promotion Institution, has supported the development of standard and systemic TKM-based health promotion programs since 2014 [8]. Since then, eight standard programs for infants, adolescents, pregnant women, adults, the elderly, and disadvantaged groups have been developed and implemented in community health centers nationwide [8]. This study aimed to develop and evaluate a standard government-supported TKM-based health promotion program for disadvantaged children.

## Methods

### Study design

A quasi-experimental, non-equivalent, prospective study involving children from a community children’s center (CCC) in South Korea was designed to evaluate the effectiveness of a standard TKM-based health promotion program for disadvantaged children.

### Participants and data collection

The participants of this study were disadvantaged children belonging to CCCs in South Korea. A CCC is a welfare daytime facility for children from socially disadvantaged families, which provides comprehensive childcare services ranging from basic care and education to leisure activities [15]. The requirements for using CCCs are being ‘under 18 years of age in need of protection.’ Need of protection is determined based on household income and need for care. Household income must be less than 100% of the median income, and the need for care is indicated by a single-parent family, family with disabilities, grandparents as primary caregivers, etc [16]. Other detailed requirements for children may differ in each community. Since its enactment in 2004, more than 100,000 children have been cared for daily in more than 4,000 CCCs across South Korea [17]. However, most services provided in CCCs are limited to care and education, leaving little support for essential health promotion services required for children [18].

Sixteen CCCs in Busan and Yangsan, South Korea, were recruited as research institutions by convenience sampling. To assign these 16 CCCs to either the intervention or control group, we paired two geographically close and similar-sized (number of children and teachers) centers and created a total of eight pairs. Then, the two CCCs included in each matched pair were randomized to the intervention or control group using a coin flipper.

From July to October 2016, the children from the eight intervention CCCs were provided with a 12-week TKM-based health promotion program, along with CCC basic services (care, education, and leisure activities). Children from the eight control CCCs were included in the waitlist control group and did not undergo the program; they were provided with only CCC basic services. After this study was all completed, considering ethical aspects, the same health promotion program was provided to children in the control group; however, this data was not included in the analysis.

Data for analyzing the program effectiveness were collected through self-administered pre- and post-surveys conducted on paper with the legal representatives (caregivers) of the children. The pre-survey was conducted prior to implementing the program and consisted of the children’s demographic characteristics, medical use, and health status (Additional file 1). The post-survey was conducted after the 12-week program was completed, which excluded demographic questions from the pre-survey (Additional file 2). The same questionnaires were administered to both the intervention and control groups.

### Intervention: standard TKM-based health promotion program

In this study, the standard TKM-based health promotion program was developed in accordance with previous guidelines [19] to reflect health-related issues and the needs of disadvantaged children in primary care settings. In the first phase, researchers visited the participating CCCs to conduct in-depth interviews and surveys with CCC teachers and children and identify major health issues and related environmental status. In the second phase, the program was drafted to prioritize health issues and needs. In the third phase, the draft was revised based on expert advice and the final program was developed.

TKM, the central theory of the program, has long emphasized health promotion and disease prevention from the perspective of Yang-Saeng (養生) and Mi-byeong (未病). Yang-Saeng means ‘taking care one’s health,’ and promoting health in daily life based on food, clothing, shelter, and the external environment [20]. Mi-byeong means that it is ‘not yet an illness’, which refers to a health or sub-health condition. This demonstrates the perspective of TKM, which values prevention before the onset of the disease [21].

The program participants included CCC teachers, children, their caregivers, and TKM doctors. Eight TKM doctors in private clinics participated voluntarily. To minimize variations caused by the difference in performance capabilities among TKM doctors, we prepared a program manual, standard consultation form, and educational materials, and held a pre-workshop for TKM doctors. Each TKM doctor was assigned to each intervention CCC. They provided comprehensive health promotion services based on the TKM throughout the 12-week program. Specifically, for ‘Medical examination and counselling’ and ‘Education and practice’ TKM doctors visited the CCCs four times within the 12-week program. The standard TKM-based health promotion program consists of four parts.

#### Part 1. Medical examination and counselling

Medical examination and counselling was conducted in cooperation with CCC teachers, children’s caregivers, and TKM doctors. Basic medical examination data were collected by CCC teachers and the children’s caregivers. CCC teachers compiled information collected through ‘Daily management’ (Part 3) and ‘Health monitoring’ (Part 4). The children’s caregivers filled out the checklist for TKM diagnosis (Additional file 3). The checklist was based on children’s health examination questionnaire [22], which was developed by the Korean Oriental Pediatrics Association using five-organ theory [23]. This is a method of inferring the internal health level of children through weakness and firmness revealed outside the children’s body. TKM doctors compiled the reported information and comprehensively examined the children.

In addition, TKM doctors conducted counselling to promote children’s health and prevent disease. They classified children’s health patterns based on the Sa-sang constitution theory of TKM, thereby inferring their general health vulnerabilities and strengths [24].

#### Part 2. Education and practice

The TKM doctor visited the CCC to provide regular education for children and CCC teachers. Regular education consisted of hygiene management, healthy habits in the summer season, growth promotion gymnastics, and allergic disease prevention. In addition, video materials were provided so that children could take care of their health, both at the CCC and at home. The CCC teacher ensured that the children completed their regular education and assisted them in smoothly performing the healthcare activities within the CCC.

#### Part 3. Daily management

Children with early respiratory, digestive, ophthalmological, otolaryngological, and dermatological symptoms were treated by the CCC teacher with in-house medicine, with the children’s caregivers’ consent and consultation with a TKM doctor. The in-house medicines consisted of herbal medicine-based extracts, syrup-type fever reducer, and ointment. These over-the- counter medicines are manufactured by pharmaceutical companies and can be used for mild digestive, cold, and dermatological symptoms.

#### Part 4. Health monitoring

The CCC teacher checked the attendance status of the children at the CCC and whether the children had abnormal physical conditions that required medical examination or management. The CCC teacher also assisted the children with filling out their health notebooks. Children self-reported data on health management activities by completing data on their health status and how they performed the healthcare activities in notebooks. The information from the notebooks was also used as a reference by TKM doctors during ‘Medical examination and counselling’ (Part 1). The TKM doctor monitored children’s health online or telephonically and provided constant advice.

### Variable

#### Outcome variables

Indicators that could reflect children’s medical use and health status were set as the outcome variables. Information on medical use and health status was collected through pre- and post-surveys by the children’s caregivers.

Medical use was assessed based on the number of outpatient visits. Health status was measured through the number of absences and lateness/early leaves due to health, number of infectious symptoms, EuroQol-5D (EQ-5D), and EQ-visual analog scale (EQ-VAS).

To minimize recall bias caused by the self-measurement of caregivers, we specified the disease and range of the measurement period. Diseases were limited to respiratory, digestive, ophthalmological, otolaryngological, and dermatological diseases, which have a large degree of incidence and clinical significance in children [25–27]. And, the number of outpatient visits, absences, and lateness/early leave within the last month(e.g. “Has your child ever received outpatient treatment for respiratory problems?”—in the last month); the number of infectious symptoms within the last two weeks(e.g. “Please check all the symptoms your child had” —in the last two weeks); and the EQ-5D and EQ-VAS based on the day of each survey(e.g. “Please mark an X on the below scale to indicate how your child’s health is TODAY.”) were only included in the calculation. These periods were set considering their consistency with Park et al. [28]. The EQ-5D index was calculated using the method proposed by the Korea Centers for Disease Control and Prevention [29].

#### Covariates

Covariates were set up to control upstream factors [30] that are expected to affect children’s medical use and health status [18, 31, 32], in addition to the intervention program.

A total of eight covariates were set in a dummy format, including sex, age, having sibling(s), main caregiver, family type, household income, maternal education level, and past medical history. These were selected from among the basic characteristics variables of children presented in Table 1, and the interconnection effects between covariates were minimized based on pie coefficients. Covariate data were described without mapping to any specific table column.

### Statistical analysis

All statistical analyses were performed using R studio version 1.3.1056 (R studio, Boston, MA, USA), with a significance level of 0.05 and a two-tailed confidence interval of 95%.

Due to the nature of the survey, missing values may occur due to unit or item non-response. Unit non-response, in this study, may be a case of participating in a pre-survey but not in a post-survey. Item non-responses is a case of participating surveys but not responding to some questionnaire items. In this study, all missing values were not imputed, only the data actually collected from the pre- and post-surveys were included in the analysis. Hence, we compared the case of ‘response’ and ‘non-response’ to check for bias induced by non-response in the intervention and control groups, respectively.

Statistical analyses were performed as follows:

- First, for the basic characteristics of children, we conducted a descriptive analysis and homogeneity test of the two groups using chi-square and Fisher’s exact tests.
- Second, the changes in medical use and health status of the children in each group before and after the program was applied to the intervention group were compared using the t-test.
- Third, we evaluated the effects of the program by controlling for covariates, using a difference-in-differences (DID) model with regression analysis [33]. All eight covariates presented in “Covariates” were controlled for, and the effect calculated through this was interpreted as the main effect value. However, in order to confirm the reliability of the effect, a sensitivity analysis was conducted. In sensitivity analysis, among the eight covariates, only those with significant differences between the two groups in the pre-survey were controlled. For the regression analysis of DID model, the number of outpatient visits, absences, lateness/early leaves, and infectious symptoms were based on the zero-inflated negative binomial (ZINB) regression model. The ZINB regression model was fitted on the assumption that the outcome variables were over-dispersed and zero-inflated [34, 35]. The estimates from the DID model with ZINB regression analysis are presented as an incident rate ratio (IRR), the exponentially multiplied value of the regression coefficient (as $${e}^{regression coefficient})$$. Moreover, the ED-5D and ED-VAS were analyzed using the tobit regression model because they were censored data [36].
  $$\begin{array}{c}{y}_{i}=\mathrm{ \alpha }+ {\upbeta }_{1}\left(\mathrm{group}:\mathrm{time}\right)+ {\upbeta }_{2}\mathrm{group}+ {\upbeta }_{3}\mathrm{time }+ {\upbeta }_{4}{X}_{j}+\upvarepsilon \\ IRR= {e}^{regression coefficient = {e}^{\upbeta }}\end{array}$$
- ${y}_{i}:$ Outcome variables – outpatient visit, absence, lateness/early leave, infectious symptom, EQ-5D, EQ-VAS
  $$\mathrm{\alpha }$$: Intercept
  $$\upbeta$$: Regression coefficient
  group: Control group=0, Intervention group=1
  time: Before the program=0, After the program=1
  ${X}_{j}$: Covariates – sex, age, having sibling(s), main caregiver, family type, household income, maternal education level, and past medical history
  $$\upvarepsilon$$: Error
  $$\mathrm{e}$$: Exponential
  ${IRR}_{{\beta }_{1}}(={e}^{{\beta }_{1}})$: Program effects on the outpatient visit, absence, lateness/early leave, infectious symptom
  Fourth, a goodness-of-fit test was conducted to assess the suitability of the ZINB regression model and to ensure the reliability of the analysis. The over-dispersion assumption was evaluated using the likelihood-ratio test [37]. The zero-inflated assumption was evaluated using both the Vuong test [38] and Akaike’s information criterion (AIC) [39], considering the limitations of the Vuong test [40].

## Results

**Fig. 1:**
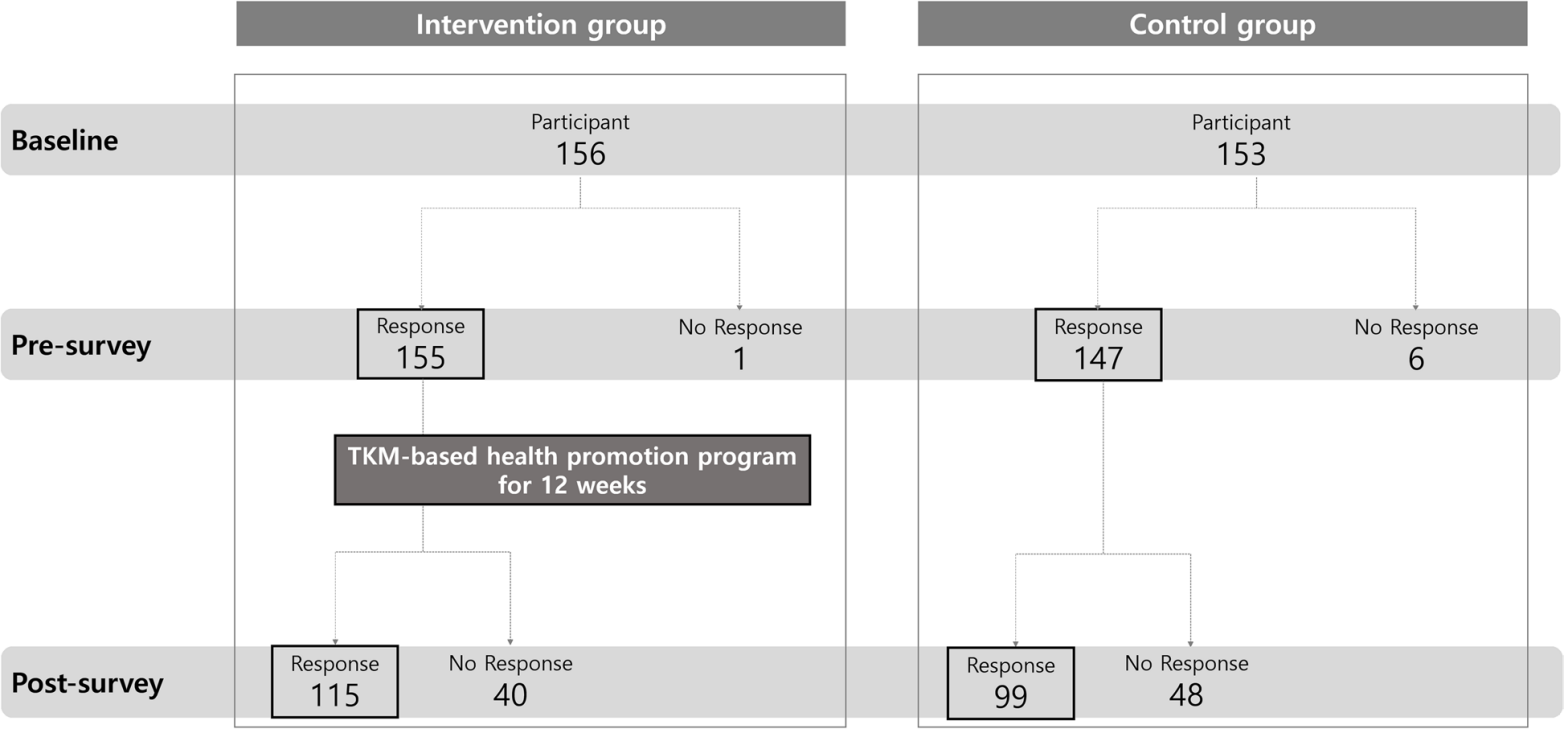
The result of participation and data collection

At baseline, 309 children (156 and 153 in the intervention and control groups, respectively) were enrolled in the study. In total, 155 (99.4%) and 147 (96.1%) children in the intervention and control groups, respectively, responded to the pre-survey. In the post-survey, 115 (73.7%) and 99 (64.7%) children in the intervention and control groups, respectively, responded. Seven children (one and six in the intervention and control groups, respectively) were excluded from the analysis because their questionnaires were not returned in the pre-survey and there was no sociodemographic information available for analysis (Fig. 1).

Regarding the decrease in the response rate in the post-survey, we compared children who completed the pre- and post-survey and children who dropped out after the pre-survey (unit non-response) in the intervention and control groups, respectively. We found that their basic characteristics were generally homogeneous and that the bias due to unit non-response would not be large (Additional file 4). In the case of item non-responses, the proportion was <5% of the total response; thus, the bias due to the missing value can be ignored [41].

### Basic characteristics and homogeneity of children (Table 1)

The basic characteristics of the two groups were compared pre- and post-survey. Owing to the characteristics of this study conducted at CCCs in the real world, it was necessary to check the heterogeneity between groups. In the pre-survey, the intervention group had a significantly higher number of children with two parents (*p* = 0.03) than did the control group. In the post-survey, the intervention group had a significantly higher number of children with a past medical history (*p* = 0.02) and present illness (*p* = 0.04) than the control group (Table 1).

**Table 1:** Basic characteristics and homogeneity of children

	**Pre-survey (*n* = 302)**			**Post-survey (*n* = 214)**		
	Intervention group (*n* = 155)	Control group (*n* = 147)	*p*-value^1^	Intervention group (*n* = 115)	Control group (*n* = 99)	*p*-value^1^
**Sex**						
Male	49.00%	47.60%	0.81	52.20%	47.50%	0.49
Female	51.00%	52.40%		47.80%	52.50%	
**Age**						
Mean(SD)	10.45(1.60)	10.50(1.72)	0.76	10.41(1.67)	10.37(1.74)	0.88
≥10 years	69.00%	70.10%	0.85	66.10%	66.70%	0.93
<10 years	31.00%	29.90%		33.90%	33.30%	
**Having sibling(s)**						
Yes	85.50%	87.40%	0.64	86.70%	86.60%	0.98
No	14.50%	12.60%		13.30%	13.40%	
**Main caregiver**						
Mother	74.70%	72.80%	0.71	75.70%	71.70%	0.51
Others	25.30%	27.20%		24.30%	28.30%	
**Family type**						
Two parents	78.90%	68.00%	0.03*	80.50%	70.70%	0.09
Others	21.10%	32.00%		19.50%	29.30%	
**Insurance type**						
NHI	86.10%	84.20%	0.65	84.00%	85.10%	0.82
Not NHI	13.90%	15.80%		16.00%	14.90%	
**Household income**						
≥3000$	32.00%	32.60%	0.91	30.30%	36.20%	0.37
<3000$	68.00%	67.40%		69.70%	63.80%	
**Paternal education level**						
Bachelor’s degree or above	54.20%	59.80%	0.36	51.60%	56.30%	0.52
High school or less	45.80%	40.20%		48.40%	43.70%	
**Maternal education level**						
Bachelor’s degree or above	54.70%	50.40%	0.49	54.40%	40.30%	0.06
High school or less	45.30%	49.60%		45.60%	59.70%	
**Paternal employment**						
Yes	95.40%	95.20%	0.94	93.80%	95.30%	0.64
No	4.60%	4.80%		6.20%	4.70%	
**Maternal employment**						
Yes	68.60%	69.10%	0.93	71.20%	68.30%	0.67
No	31.40%	30.90%		28.80%	31.70%	
**Having past medical history**						
Yes	54.80%	43.50%	0.05	56.50%	40.40%	0.02*
No	45.20%	56.50%		43.50%	59.60%	
**Having present illness**						
Yes	25.80%	19.00%	0.16	31.30%	19.20%	0.04*
No	74.20%	81.00%		68.70%	80.80%	

*Abbreviations*: *NHI* National Health Insurance, *SD* Standard deviation

^*^p < 0.05, statistically significant

^1^This column presents *p-values* of chi-square tests for binomial variables and independent t-tests for continuous variables

### Changes in medical use and health status of children (Table 2, Fig. 2)

**Table 2:** Changes in medical use and health status of children

	**Intervention group (** ***n *** **= 115)**				**Control group (** ***n *** **= 99)**			
	Pre-survey	Post-survey	Difference^1^	*p*-value^2^	Pre-survey	Post-survey	Difference^1^	*p*-value^2^
**Outpatient visits**	0.84	0.83	-0.01	0.88	0.65	1.98	1.33	0.05
**Absences**	0.42	0.07	-0.35	0.02*	0.19	0.06	-0.13	0.17
**Lateness/early leaves**	0.10	0.09	-0.02	0.77	0.11	0.08	-0.03	0.60
**Infectious symptoms**	1.59	1.63	0.03	0.72	1.09	1.35	0.26	0.15
**EQ-5D**	0.96	0.94	-0.02	0.04*	0.97	0.96	0.00	0.80
**EQ-VAS**	86.74	90.01	3.27	0.02*	87.09	85.72	-1.38	0.28

*Abbreviations*: *EQ-5D* EuroQol-5D, *EQ-VAS* EuroQol-visual analog scale

^*^p < 0.05, statistically significant

^1^Difference = Post-survey – Pre-survey

^2^This column presents *p-values* of dependent t-tests for children who completed the pre- and post-surveys in the intervention and control groups, respectively

**Fig. 2:**
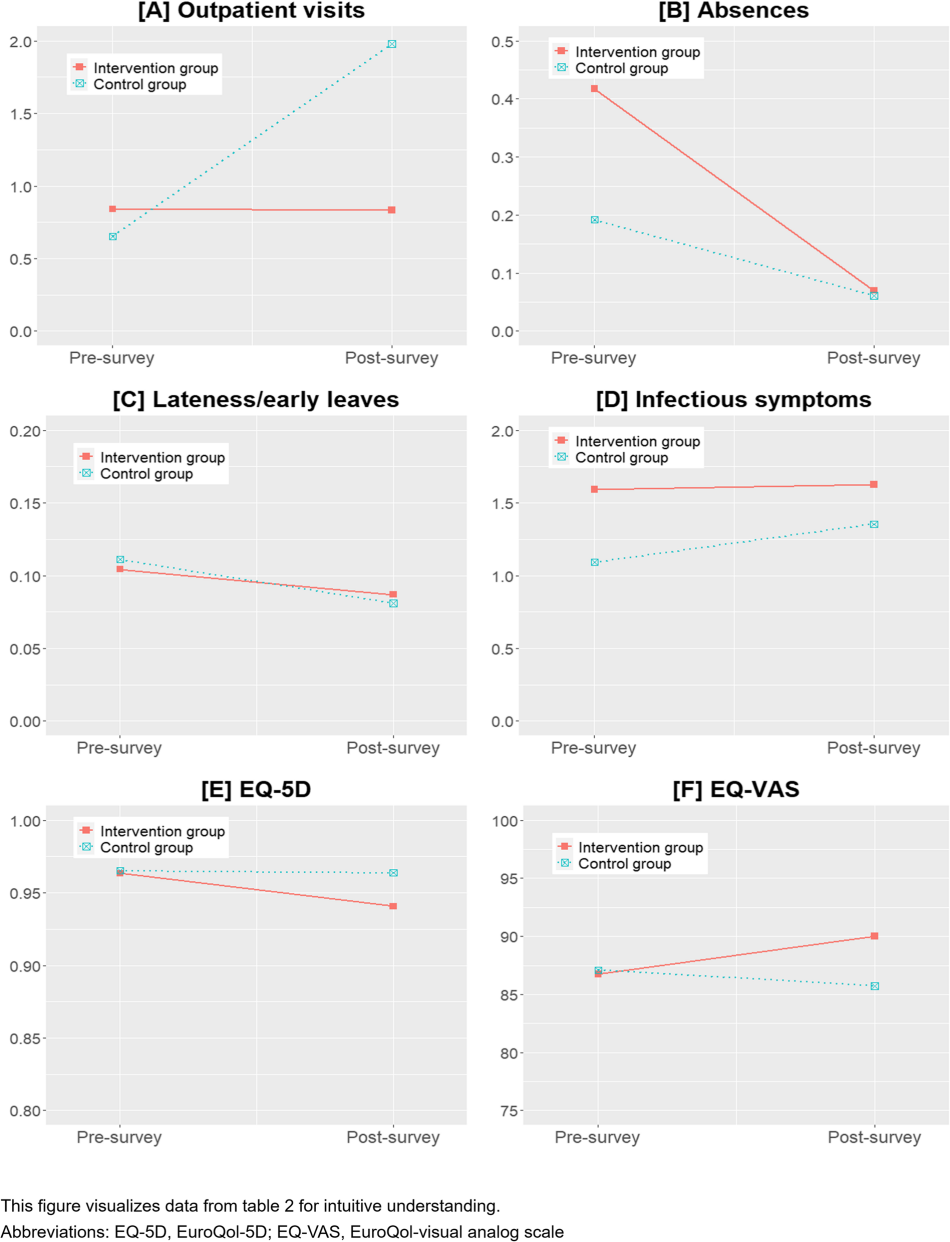
Change in Medical use and health status of children

Changes in the medical use and health status of the children in the two groups were compared before and after the program was applied to the intervention group.

The number of outpatient visits decreased by 0.01 in the intervention group but was not significant; in the control group, it increased by 1.33 and was not significant. The number of absences decreased by 0.35 and 0.13 in the intervention and control groups, respectively, but the difference was significant only in the intervention group. The number of lateness/early leaves decreased by 0.02 and 0.03 in the intervention and control groups, respectively, but the difference was not significant. The number of infection symptoms increased by 0.03 and 0.26 in the intervention and control groups, respectively, but the difference was not significant. The EQ-5D decreased by 0.02 in the intervention group and was significant, but it did not change in the control group. The EQ-VAS score in the intervention group increased by 3.27 and was significant, and that in the control group decreased by 1.38, but the difference was not significant.

### Effectiveness of the program (Table 3)

**Table 3:** Effectiveness of the program

	**Main effect** ^1^				**Effect through sensitivity analysis** ^2^			
	**IRR** ^3^	β^4^	SE	*p*-value^5^	**IRR** ^3^	β^4^	SE	*p*-value^5^
**Outpatient visit**	0.35	-1.04	0.48	0.03*	0.23	-1.47	0.52	0.00*
**Absence**	0.49	-0.72	0.97	0.46	0.03	-3.58	1.47	0.01*
**Lateness / early leave**	0.53	-0.63	1.27	0.62	0.43	-0.84	1.06	0.43
**Infectious symptom**	0.86	-0.15	0.23	0.51	0.73	-0.32	0.24	0.18
**EQ-5D**	-	-0.02	0.01	0.19	-	-0.02	0.01	0.15
**EQ-VAS**	-	8.00	3.26	0.01*	-	5.39	2.99	0.07

*Abbreviations*: *IRR* Incidence rate ratio, *β* Regression coefficient of (group:post), *SE* Standard error, *EQ-5D* EuroQol-5D, *EQ-VAS* EuroQol-visual analog scale

^*^p < 0.05, statistically significant

^1^Main effect analysis: Eight covariates were adjusted

^2^Sensitivity analysis: Among eight covariates, only those with significant differences between the two groups in the pre-survey, which was ‘family type’, was adjusted

^3^The IRR is an exponentially multiplied value of regression coefficients. Among them, this represents$${e}^{regression coefficient of group:time}$$

^4^The β is a regression coefficient. Among them, this represents the coefficient of interaction term (group:time) in regression analysis

^5^This column presents *p-values* of difference-in differences model with regression analysis. The number of outpatient visits, absences, lateness/early leaves, and infectious symptoms were based on the zero-inflated negative binomial regression analysis; the ED-5D and ED-VAS were based on the tobit regression analysis

Through DID analysis with a regression model, the main effectiveness of the program was analyzed by controlling for eight covariates.

The number of outpatient visits was significantly lower (by 65%) in the intervention group than in the control group (IRR = 0.35, *p* = 0.03). The number of absences, lateness/early leave, and infectious symptoms decreased by 51% (IRR = 0.49), 47% (IRR = 0.53), and 14% (IRR = 0.86), respectively, in the intervention group compared to that in the control group; however, the differences were not significant. For EQ-5D, the program had a negative effect, but it was not significant. In the EQ-VAS, the program had a positive and significant effect.

### Sensitivity analysis (Table 3)

Among the eight covariates, the sensitivity analysis was performed by controlling only the ‘family type,’ for which covariate showed a significant difference between both groups in the pre-survey.

The number of outpatient visits was significantly lower (by 77%) in the intervention group than in the control group (IRR = 0.23, *p* = 0.00). The number of absences in the intervention group was significantly lower (by 97%) than in the control group (IRR = 0.03, *p* = 0.01). The number of lateness/early leave and infectious symptoms decreased by 57% (IRR = 0.43) and 27% (IRR = 0.73), respectively, in the intervention group compared to that in the control group; however, the differences were not significant. In the EQ-5D, the program application had a negative effect, but it was not significant. In the EQ-VAS, the program application had a positive effect, but it was not significant.

### Goodness of fit test (Additional file 5)

The likelihood-ratio test revealed that the likelihood of a negative binomial model that reflected overdispersion assumptions was greater than that of a poisson model that did not reflect overdispersion assumptions.

Based on the negative binomial models, the suitability of the zero-inflated assumption was confirmed through the Vuong test. The ZINB model reflecting zero-inflated assumptions was suitable for the number of outpatient visits and absences, but there was no significant difference in the number of lateness/early leaves and infectious symptoms. However, additional AIC analysis showed that it was more appropriate to reflect the zero-inflated assumptions in all four outcome variables.

## Discussion

In this study, we developed a TKM-based health promotion program as a strategy to improve disadvantaged children’s health problems, and evaluated its effectiveness based on the medical use and health status of children in CCCs.

The program significantly reduced the number of outpatient visits both in the main analysis, and in the sensitivity analysis. This is consistent with that reported in a study by Park et al. [28], which evaluated a TKM-based healthcare program for preventing infectious diseases in infants. This may suggest that providing TKM doctor visits to CCCs and herbal medication could reduce the number of outpatient visits. However, considering this program as a substitute for visiting medical institutions could underestimate its effectiveness, given that it did not directly diagnose or treat children’s diseases, but instead conducted health monitoring and prevention activities within the child’s living environment. It must also be noted that we cannot conclude whether the program effect on outpatient visits is due to the increase of the control group (Fig. 2), because Fig. 2 (Changes in medical use and health status of children (Table 2, Fig. 2)) only shows mean values not excluding the influence of covariates.

Although the number of absences, lateness/early leaves, and infectious symptoms in the intervention group tended to be lower than those in the control group, there was no significant difference between the groups in the main effect analysis. This result may be attributed to several factors. First, the intervention period of this study was from July to September, and in South Korea, August is the vacation period. Therefore, it may have been difficult to demonstrate a difference in attendance or lateness/early leaves due to the small number of school days. In addition, regarding infectious symptoms, it may have been difficult to detect a difference between the two groups because South Korea has a good sanitary environment with few digestive infections; moreover, respiratory infections generally occur frequently after October in South Korea.

In the intervention group, the number of absences decreased significantly in the sensitivity analysis, and EQ-VAS increased significantly in the main effect analysis. However, it should be interpreted conservatively as the result somewhat varies compared to outpatient visits.

In analyzing program effectiveness, the individual effects of each part of the program were not analyzed separately. This complements the purpose of this study, which was to develop a comprehensive packaged service program, rather than to prove the effects of a single intervention for health promotion.

The DID used as the analysis model for this study are popular in empirical research to estimate the causal effect of certain policy interventions or changes that cannot affect everybody simultaneously and in the same way [33]. This model not only controls the unmeasured time-varying factors (trend effect), but also offsets the heterogeneity between the intervention and control groups based on the assumption that they are time invariant. However, even in DID, when setting up a control group, the propensity score matching method is used or multiple control groups are included in consideration of internal validity. In this study, children’s demographic information was collected as bivariate, which limited the accuracy of the propensity score matching. In addition, it was difficult to establish multiple control groups because local communities and CCCs had different opinions and circumstances regarding participation.

To compensate for these problems, we conducted a homogeneity test and referred the results to the program effects. Homogeneity tests showed some heterogeneity among the participating children. However, it is unlikely that this would have overestimated the program’s effectiveness. According to the homogeneity test, the proportion of children with two-parent family types was significantly higher in the intervention group than in the control group in the pre-survey. In the post-survey, the proportion of children with a past medical history and present illness was significantly higher in the intervention group than in the control group. This indicates that the effectiveness of the program was judiciously measured because intervention group in the post-survey included more children with poor underlying health status than the control group. In addition, the higher proportion of children with present illness in the post-survey of the intervention group can be interpreted as inducing preventive effects through early disease diagnosis via this study program.

This TKM-based health promotion program presents a comprehensive and systemic health promotion strategy comprising medical examination and counselling, education and practice, daily management, and health monitoring. CCCs in South Korea are usually small centers composed of a director and two or three childcare teachers; moreover, they cannot have their own medical personnel and facilities. In this program, medical examinations and consultations by visiting TKM doctors helped to determine the children’s current health and developmental levels. In addition, this program was intended to increase children’s health knowledge and interest in health through periodic health education and health monitoring using a self-reported health diary. To facilitate this multi-faceted program, both visiting TKM doctors and CCC teachers and caregivers were assigned cooperative roles. This is partly consistent with the recent trend of emphasizing the partnership of diverse personnel in child healthcare [42]. However, due to the spatial limitations of the research environment of CCCs, the scope of caregivers’ roles was limited to providing consent and filling questionnaires for children’s medical use and health status. In the future, it will be necessary to strengthen education or performance to increase the role of child caregivers through subsequent program development.

Traditional, complementary, and alternative medicine (T&CAM) has been used to treat children’s health problems in many medical centers worldwide. In the United States, the Integrative Therapies Team Program of Boston Children’s Hospital has been established to provide massage therapy, guided imagery, reiki, acupuncture, expressive arts, and yoga [43]. This team program is also available in several institutions in various areas such as Minnesota, Philadelphia, Colombia, Utah, and Orange County [44–48]. In Germany, the Integrative Pediatrics project (Integrative Pädiatrie Projekt) was conducted from 2015 to 2017 in three medical pediatric hospitals [49]; and, reportedly, 80% of caregivers in the project want to use T&CAMs in hospitals for the treatment of their children [50]. In Switzerland, the Integrative Pediatrics Center (Center de pédiatrie intégrative) has been providing homeopathic medicine, herbal medicine, acupuncture, and cupping for pediatric diseases since 2015 [51]. In particular, the Department of Pediatric Hematologic Oncology at Bern University Hospital has collaborated with complementary alternative medical institutions in the university since 2010 to provide T&CAM treatment for children hospitalized for cancer [52].

The above cases show the possibility of using T&CAM for the treatment of pediatric diseases within medical institutions. However, in the real-world community environment, the application to disease prevention and health promotion was relatively small, and the use of quantified indicators was insufficient to evaluate its effectiveness. Thus, this study is meaningful because it quantitatively evaluates the effectiveness of T&CAM in disease prevention and health promotion in a real-world, community setting.

One potential limitation of this study is that since the participation of TKM doctors in private clinics was voluntary, we could not control for their abilities or fields of expertise. This was because many childcare facilities in South Korea had difficulty securing medical professionals [53–55]. If this program is extended to the local community, regarding cost-effectiveness, it would be more appropriate to use TKM doctors hired by community health centers rather than those who run private clinics.

Another limitation was that the 12-week intervention period was relatively short to determine the disease prevention effect of the health promotion program, and long-term follow-up after the intervention period was not performed. Third, the CCCs participating in the study were recruited through convenience sampling rather than random sampling. Thus, strict control over the representativeness of the study subjects was difficult, suggesting that the generalizability of this study’s results must be approached conservatively. However, considering the number of CCCs and the operational status of each CCC within the geographic scope of the study, it was difficult for researchers to randomly select CCCs to participate in the study. And, in the case of group allocation, efforts were made to minimize bias when evaluating program effects using random allocation. Forth, although the beneficiaries of the study program were children, data for measuring the results were reported by their caregivers. The EQ-5D index and VAS score were reported indirectly by the children’s caregiver, not reported directly by the children using EQ-5D-Y.

Lastly, as it is rare for child caregivers to visit CCCs, it was difficult for researchers to actively and directly recommend caregivers to participate in pre- and post-surveys for data collection.

Despite these limitations, it was possible to observe some positive outcomes. It is necessary to consider a longer period of intervention and follow-up observations when designing future studies.

## Conclusions

This study identified the health-related problems and environments of disadvantaged children in South Korea, developed a standard TKM-based health promotion program, and evaluated its effectiveness. We identified the opportunity for a comprehensive health promotion program based on TKM regarding medical use and health status of disadvantaged children.

Further consideration is necessary for developing systems using TKM as part of various social approaches to improve the health of disadvantaged children. Thus, a long-term, successive, and large-scale follow-up study is needed for a systematic and objective evaluation.

## Supplementary Information

**Additional file 1.** A questionnaire of pre-survey.

**Additional file 2.** A questionnaire of post-survey.

**Additional file 3.** The checklist for TKM diagnosis.

**Additional file 4.** Basic characteristics and homogeneity of children based on whether they completed the pre- and post-survey or only the pre-survey.

**Additional file 5.** Goodness of fit test results.

## Data Availability

The datasets generated in the current study are available from the corresponding author upon reasonable request.

## Abbreviations

TKM: Traditional Korean Medicine
CCC: Community children’s center
EQ-5D: EuroQol-5D
EQ-VAS: EuroQol-visual analog scale
DID: Difference-in-differences
ZINB: Zero-inflated negative binomial
IRR: Incident rate ratio
AIC: Akaike’s information criterion
T&CAM: Traditional & Complementary and Alternative Medicine
