# Supplementary material for: Evaluation of the effectiveness of the standard traditional Korean medicine-based health promotion program for disadvantaged children in South Korea
Source: BMC Complement Med Ther. 2022 Jun 26;22:175. doi: 10.1186/s12906-022-03634-w (PMC9233805; doi:10.1186/s12906-022-03634-w)
Supplement: Supplementary file 1 — Additional file 1. A questionnaire of pre-survey. [file 12906_2022_3634_MOESM1_ESM.docx]

Additional file 1: A questionnaire of pre-survey

A questionnaire on the general characteristics and health status of children (1st: pre-survey)

| * For each question, please check(√) or fill in the blank. |
| --- |

| Ⅰ. Children’s basic information |
| --- |

1. Child’s gender □ ① Male □ ② Female

2. Child’s age _____ years old

3. Child’s birth date YYYY - MM - DD

4. Child’s siblings e.g., First, second, and third of e.g., One, two, and three

5. What is your relationship with the child?

□ ① mother □ ② father □ ③ grandparents □ ④ other ( )

6. How long has your child attended this Community Children’s Center?

□ ① < 1 year □ ② ≥1, <3 years □ ③ ≥ 3 years

7. Who is the main caregiver of the child?

□ ① mother □ ② father □ ③ grandmother

□ ④ grandfather □ ⑤ other ( )

8. Has your child ever been diagnosed or treated by a doctor (or traditional Korean medicine doctor) in the past?

□ ① No (→ to NO. 9)

□ ② Yes (→ to NO. 8-1)

8-1. What was(were) the disease(s)? ※ multiple choices available

□ ① Pneumonia □ ② Bronchitis □ ③ Tympanitis

□ ④ Allergic rhinitis □ ⑤ Sinusitis □ ⑥ Gastroenteritis.

□ ⑦ Atopic dermatitis □ ⑧ Asthma □ ⑨ Conjunctivitis

□ ⑩ Other ( )

9. Is your child currently being treated or has any problematic disease?

□ ① No (→ to NO. 10)

□ ② Yes (→ to NO. 9-1)

9-1. What is(are) the disease(s)? ※ multiple choices available

□ ① Pneumonia □ ② Bronchitis □ ③ Tympanitis

□ ④ Allergic rhinitis □ ⑤ Sinusitis □ ⑥ Gastroenteritis.

□ ⑦ Atopic dermatitis □ ⑧ Asthma □ ⑨ Conjunctivitis

□ ⑩ Other ( )

| Ⅱ. Children’s medical use (in the last 1 month) |
| --- |

10. Has your child ever received outpatient treatment for respiratory problems?

(Respiratory problems include colds, tonsillitis, bronchitis, pneumonia, rhinitis, etc.)

□ ① No (→ to NO. 11)

□ ② Yes (→ to NO. 10-1)

10-1. How many days did your child visit for outpatient treatment? ____ days

10-2. What was(were) the disease(s)? ※ multiple choices available

□ ① Cold(acute nasopharyngitis) □ ② Tonsillitis □ ③ Bronchitis.

□ ④ Pneumonia □ ⑤ Rhinitis □ ⑥ Other( )

10-3. Did your child receive hospital treatment for the above disease(s)?

□ ① No (→ to NO.11) □ ② Yes (→ to NO.10-4)

10-4. How many days was your child hospitalized? ____ days

11. Has your child ever received outpatient treatment for digestive problems?

(Digestive problems included indigestion, gastritis, and enteritis.)

□ ① No (→ to NO. 12)

□ ② Yes (→ to NO. 11-1)

11-1. How many days did your child visit for outpatient treatment? ____ days

11-2. What was(were) the disease(s)? ※ multiple choices available

□ ① Indigestion □ ② Gastritis

□ ③ Enteritis □ ④ Other ( )

11-3. Did your child receive hospital treatment for the above disease(s)?

□ ① No (→ to NO.12) □ ② Yes (→ to NO.11-4)

11-4. How many days was your child hospitalized? ____ days

12. Has your child ever received outpatient treatment for ophthalmological, otolaryngological, and dermatological problems?

(Digestive problems include conjunctivitis, dermatitis, hives, tympanitis, etc.)

□ ① No (→ to NO. 13)

□ ② Yes (→ to NO. 12-1)

12-1. How many days did your child visit for outpatient treatment? ____ days

12-2. What was(were) the disease(s)? ※ multiple choices available

□ ① Conjunctivitis □ ② Dermatitis □ ③ Hives.

□ ④ Tampanitis □ ⑤ Other ( )

12-3. Did your child receive hospital treatment for the above disease(s)?

□ ① No (→ to NO.13) □ ② Yes (→ to NO.12-4)

12-4. How many days was your child hospitalized? ____ days

| Ⅲ. Children’s daily activities due to health problems (in the last 1 month) |
| --- |

13. Has your child ever been absent due to health problems?

□ ① No

□ ② Yes ⇨ If yes, how many days? _______ days

14. Has your child ever been late or leave early due to health problems?

□ ① No

□ ② Yes ⇨ If yes, how many days? _______ days

| Ⅳ. Children’s common symptoms (in the last 2 weeks) |
| --- |

15. Please check all the symptoms your child had. ※ multiple choices available

□ ① Fever (above 38℃) □ ② Cough □ ③ Sneezing

□ ④ Runny nose □ ⑤ Stuffy nose □ ⑥ Stomachache

□ ⑦ Diarrhea □ ⑧ Vomiting □ ⑨ Earache

□ ⑩ Ear oozing □ ⑪ Itchy eyes □ ⑫ Hyperemia

□ ⑬ Eye mucus □ ⑭ Rash (e.g., atopic dermatitis, miliaria)

□ ⑮ Epistaxis □ ⑯ None

| Ⅴ. Children’s health condition (today) |
| --- |

16. Please check that which best matches the child's health condition today for each of the following items.

16-1. Mobility (e.g., move, walk)

□ ① no problem

□ ② some problem

□ ③ severe problem

16-2. Self-care (e.g., washing, dressing)

□ ① no problem

□ ② some problem

□ ③ severe problem

16-3. Typical activities (e.g., go to school, study, and play with friends).

□ ① no problem

□ ② some problem

□ ③ severe problem

16-4. Pain / discomfort

□ ① no pain or discomfort

□ ② some pain or discomfort

□ ③ severe pain or discomfort

16-5. Anxiety / depression

□ ① no anxiety or depression

□ ② anxiety or depression

□ ③ severe anxiety or depression

17. Please mark an X on the below scale to indicate how your child’s health is TODAY.

- This line is numbered from 0 to 100
- 100 means the best health you can imagine.
-
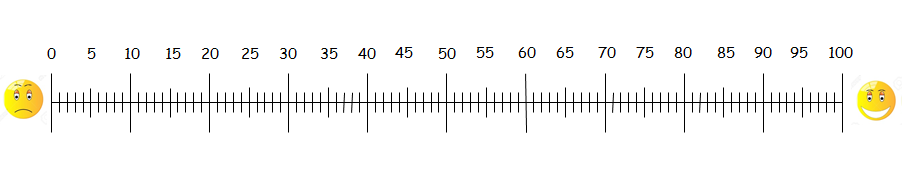
0 means the worst health you can imagine.

| Ⅵ. Children’s sociodemographic characteristics |
| --- |

| 18. Family type | □ ① Parental family (without grandparents)  □ ② Parental family (with grandparents)  □ ③ Single parent family  □ ④ Other ( ) | |
| --- | --- | --- |
| 19. Housing type | □ ① Apartment  □ ② House  □ ③ Row house  □ ④ Studio apartment  □ ⑤ Other ( ) | |
| 20. Household income | □ ① < 2,000$  □ ② ≥ 2,000$, < 3,000$  □ ③ ≥ 3,000$, < 4,000$  □ ④ ≥ 4,000$, < 5,000$  □ ⑤ ≥ 5,000$ | |
| 21. Insurance type | □ ① National Health Insurance  □ ② Medicaid  □ ③ Others ( ) | |
| 22. Parents age | Father | □ ① 20~29  □ ② 30~39  □ ③ 40~49  □ ④ over 50 |
|  | Mother | □ ① 20~29  □ ② 30~39  □ ③ 40~49  □ ④ over 50 |
| 23. Parental education level | Father | □ ① Middle school  □ ② High school  □ ③ Bachelor’s degree  □ ④ Master’s or Doctoral degree |
|  | Mother | □ ① Middle school  □ ② High school  □ ③ Bachelor’s degree  □ ④ Master’s or Doctoral degree |
| 24. Parental occupation type | Father | □ ① Administrative  □ ② Office  □ ③ Sales and service  □ ④ Manufacturing  □ ⑤ Professional  □ ⑥ Self-employed  □ ⑦ Student  □ ⑧ Housekeeping  □ ⑨ Unemployed |
|  | Mother | □ ① Administrative  □ ② Office  □ ③ Sales and service  □ ④ Manufacturing  □ ⑤ Professional  □ ⑥ Self-employed  □ ⑦ Student  □ ⑧ Housekeeping  □ ⑨ Unemployed |

** Thank you for your response **
