# Supplementary material for: Evaluation of the effectiveness of the standard traditional Korean medicine-based health promotion program for disadvantaged children in South Korea
Source: BMC Complement Med Ther. 2022 Jun 26;22:175. doi: 10.1186/s12906-022-03634-w (PMC9233805; doi:10.1186/s12906-022-03634-w)
Supplement: Supplementary file 2 — Additional file 2. A questionnaire of post-survey. [file 12906_2022_3634_MOESM2_ESM.docx]

Additional file 2: A questionnaire of post-survey

A questionnaire on the general characteristics and health status of children (2nd: Post-survey)

| * For each question, please check(√) or fill in the blank. |
| --- |

| Ⅰ. Children’s basic information |
| --- |

1. Community Children’s Center name

□ ① Seo-chang □ ② Ga-beo-na-um □ ③ Ung-sang-jung-ang

□ ④ Han-geu-ru □ ⑤ Du-re □ ⑥ Geum-saeng

□ ⑦ Boo-gok □ ⑧ Na-ne-oul

2. Child’s gender □ ① Male □ ② Female

3. Child’s name ____________

4. Is your child currently being treated for or has any problematic disease?

□ ① No (→ to NO. 5)

□ ② Yes (→ to NO. 4-1)

4-1. What is(are) the disease(s)? ※ multiple choices available

□ ① Pneumonia □ ② Bronchitis □ ③ Tympanitis

□ ④ Allergic rhinitis □ ⑤ Sinusitis □ ⑥ Gastroenteritis.

□ ⑦ Atopic dermatitis □ ⑧ Asthma □ ⑨ Conjunctivitis

□ ⑩ Other ( )

| Ⅱ. Children’s medical use (in the last 1 month) |
| --- |

5. Has your child ever received outpatient treatment for respiratory problems?

(Respiratory problems include colds, tonsillitis, bronchitis, pneumonia, rhinitis, etc.)

□ ① No (→ to NO. 6)

□ ② Yes (→ to NO. 5-1)

5-1. How many days did your child visit for outpatient treatment? ____ days

5-2. What was(were) the disease(s)? ※ multiple choices available

□ ① Cold(acute nasopharyngitis) □ ② Tonsillitis □ ③ Bronchitis.

□ ④ Pneumonia □ ⑤ Rhinitis □ ⑥ Other ( )

5-3. Did your child receive hospital treatment for the above disease(s)?

□ ① No (→ to NO.6) □ ② Yes (→ to NO.5-4)

5-4. How many days was your child hospitalized? ____ days

6. Has your child ever received outpatient treatment for digestive problems?

(Digestive problems included indigestion, gastritis, and enteritis.)

□ ① No (→ to NO. 7)

□ ② Yes (→ to NO. 6-1)

6-1. How many days did your child visit for outpatient treatment? ____ days

6-2. What was(were) the disease(s)? ※ multiple choices available

□ ① Indigestion □ ② Gastritis

□ ③ Enteritis □ ④ Other ( )

6-3. Did your child receive hospital treatment for the above disease(s)?

□ ① No (→ to NO.7) □ ② Yes (→ to NO.6-4)

6-4. How many days was your child hospitalized? ____ days

7. Has your child ever received outpatient treatment for ophthalmological, otolaryngological, and dermatological problems?

(Digestive problems include conjunctivitis, dermatitis, hives, tympanitis, etc.)

□ ① No (→ to NO. 8)

□ ② Yes (→ to NO. 7-1)

7-1. How many days did your child visit for outpatient treatment? ____ days

7-2. What was(were) the disease(s)? ※ multiple choices available

□ ① Conjunctivitis □ ② Dermatitis □ ③ Hives

□ ④ Tampanitis □ ⑤ Others ( )

7-3. Did your child receive hospital treatment for above disease(s)?

□ ① No (→ to NO.8) □ ② Yes (→ to NO.7-4)

7-4. How many days was your child hospitalized? ____ days

| Ⅲ. Children’s daily activities due to health problems (in the last 1 month) |
| --- |

8. Has your child ever been absent due to health problems?

□ ① No

□ ② Yes ⇨ If yes, how many days? _______ days

9. Has your child ever been late or left early due to health problems?

□ ① No

□ ② Yes ⇨ If yes, how many days? _______ days

| Ⅳ. Children’s common symptoms (in the last 2 weeks) |
| --- |

10. Please check all the symptoms your child had. ※ multiple choices available

□ ① Fever (above 38℃) □ ② Cough □ ③ Sneezing

□ ④ Runny nose □ ⑤ Stuffy nose □ ⑥ Stomachache

□ ⑦ Diarrhea □ ⑧ Vomiting □ ⑨ Earache

□ ⑩ Ear oozing □ ⑪ Itchy eyes □ ⑫ Hyperemia

□ ⑬ Eye mucus □ ⑭ Rash (e.g., atopic dermatitis, miliaria)

□ ⑮ Epistaxis □ ⑯ None

| Ⅴ. Children’s health condition (today) |
| --- |

11. Please check that which best matches the child's health condition today for each of the following items.

11-1. Mobility (e.g., move, walk)

□ ① no problem

□ ② some problem

□ ③ severe problem

11-2. Self-care (e.g., washing, dressing)

□ ① no problem

□ ② some problem

□ ③ severe problem

11-3. Usual activities (예: go to school, study, playing with friends)

□ ① no problem

□ ② some problem

□ ③ severe problem

11-4. Pain / discomfort

□ ① no pain or discomfort

□ ② some pain or discomfort

□ ③ severe pain or discomfort

11-5. Anxiety / depression

□ ① no anxiety or depression

□ ② anxiety or depression

□ ③ severe anxiety or depression

12. Please mark an X on the below scale to indicate how your child’s health is TODAY.

- This line is numbered from 0 to 100.
- 100 means the best health you can imagine.
- 0 means the worst health you can imagine.


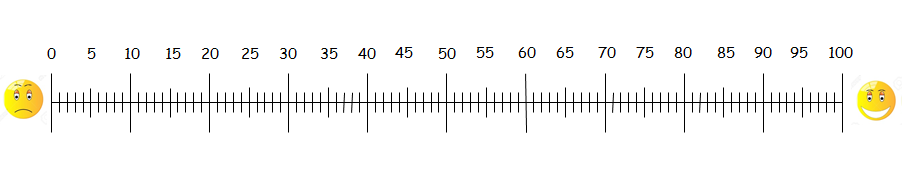


| Ⅵ. Satisfaction with the health promotion program |
| --- |

13. The contents of this program (medical examination and counselling, education, daily management, and health monitoring) met expectations.

□ ① Strongly disagree □ ② Disagree □ ③ Neutral

□ ④ Agree □ ⑤ Strongly agree

14. The three-month program implementation period was appropriate.

□ ① Strongly disagree □ ② Disagree □ ③ Neutral

□ ④ Agree □ ⑤ Strongly agree

15. Through this program, the child's ability to recognize and manage health problems independently has been improved.

□ ① Strongly disagree □ ② Disagree □ ③ Neutral

□ ④ Agree □ ⑤ Strongly agree

16. This program is effective in preventing diseases and improving children’s health.

□ ① Strongly disagree □ ② Disagree □ ③ Neutral

□ ④ Agree □ ⑤ Strongly agree

17. I am satisfied with the content and process of this program.

□ ① Strongly disagree □ ② Disagree □ ③ Neutral

□ ④ Agree □ ⑤ Strongly agree

18. After participating in the program, I think it is necessary to implement a traditional Korean medicine-based health promotion program in community children's centers.

□ ① Strongly disagree □ ② Disagree □ ③ Neutral

□ ④ Agree □ ⑤ Strongly agree

19. I am willing to participate in this program again.

□ ① Strongly disagree □ ② Disagree □ ③ Neutral

□ ④ Agree □ ⑤ Strongly agree

20. I am willing to recommend this program to others.

□ ① Strongly disagree □ ② Disagree □ ③ Neutral

□ ④ Agree □ ⑤ Strongly agree

** Thank you for your response **
