# Supplementary material for: Evaluation of the effectiveness of the standard traditional Korean medicine-based health promotion program for disadvantaged children in South Korea
Source: BMC Complement Med Ther. 2022 Jun 26;22:175. doi: 10.1186/s12906-022-03634-w (PMC9233805; doi:10.1186/s12906-022-03634-w)
Supplement: Supplementary file 3 — Additional file 3. The checklist for TKM diagnosis. [file 12906_2022_3634_MOESM3_ESM.docx]

Additional file 3: The checklist for TKM diagnosis

A checklist for traditional Korean Medicine diagnosis

(Children’s caregivers)

___________ Community Children’s Center

Child’s Name ( ) Age ( years)

※ For each question, please check(√) on the blank.

| Respiratory system | Always | Sometimes | Neutral | Rarely | Never |
| --- | --- | --- | --- | --- | --- |
| My child catches a cold often, and if (s)he does, (s)he doesn't get well. |  |  |  |  |  |
| My child often coughs at night and dawn. |  |  |  |  |  |
| My child coughs easily just by being in the cold wind or eating cold food. |  |  |  |  |  |
| My child often sneezes and has a runny and stuffy nose. |  |  |  |  |  |
| My child's tonsils become big and swollen easily. |  |  |  |  |  |
| My child experiences respiratory problems easily during the change of seasons. |  |  |  |  |  |
| My child easily develops complications such as sinusitis, otitis media, and asthma after a cold. |  |  |  |  |  |
| My child has a lot of phlegm. |  |  |  |  |  |
| My child is prone to lymph nodes and swelling. |  |  |  |  |  |
| My child often has nosebleeds. |  |  |  |  |  |
| My child's skin is often dry and itchy. |  |  |  |  |  |
| Digestive system | Always | Sometimes | Neutral | Rarely | Never |
| My child often gets indigestion. |  |  |  |  |  |
| My child often has a stomachache. |  |  |  |  |  |
| My child often has diarrhea. |  |  |  |  |  |
| My child has frequent constipation. |  |  |  |  |  |
| My child often vomits or experiences nausea. |  |  |  |  |  |
| My child often feels bloated. |  |  |  |  |  |
| My child has bad breath. |  |  |  |  |  |
| My child doesn't eat well and eats less while being picky. |  |  |  |  |  |
| My child gets motion sickness often. |  |  |  |  |  |
| My child easily feels tired, is lethargic, and inactive. |  |  |  |  |  |
| My child has no energy after eating and likes to lie down. |  |  |  |  |  |
