# Supplementary material for: Evaluation of the effectiveness of the standard traditional Korean medicine-based health promotion program for disadvantaged children in South Korea
Source: BMC Complement Med Ther. 2022 Jun 26;22:175. doi: 10.1186/s12906-022-03634-w (PMC9233805; doi:10.1186/s12906-022-03634-w)
Supplement: Supplementary file 4 — Additional file 4. Basic characteristics and homogeneity of children based on whether they completed the pre- and post-survey or only the pre-survey. [file 12906_2022_3634_MOESM4_ESM.docx]

Additional file 4: Basic characteristics and homogeneity of children based on whether they completed the pre- and post-survey or only the pre-survey

| Intervention group | | | | |
| --- | --- | --- | --- | --- |
|  | | Complete the pre- and post-surveys  (n = 115) | Complete only the pre-survey  (n = 40) | p-value^1^ |
| Sex | Male | 52.20% | 40.00% | 0.18 |
|  | Female | 47.80% | 60.00% |  |
| Age | Mean(SD) | 10.41(1.67) | 10.55(1.38) | 0.63 |
|  | ≥10 years | 66.10% | 77.50% | 0.18 |
|  | <10 years | 33.90% | 22.50% |  |
| Having sibling(s) | Yes | 86.70% | 82.10% | 0.47 |
|  | No | 13.30% | 17.90% |  |
| Main caregiver | Mother | 75.70% | 71.80% | 0.63 |
|  | Others | 24.30% | 28.20% |  |
| Family type | Two parents | 80.50% | 74.40% | 0.41 |
|  | Others | 19.50% | 25.60% |  |
| Insurance type | NHI | 84.00% | 92.10% | 0.21 |
|  | Not NHI | 16.00% | 7.90% |  |
| Household income | ≥3000$ | 30.30% | 36.80% | 0.45 |
|  | <3000$ | 69.70% | 63.20% |  |
| Paternal education level | Bachelor’s degree or above | 51.60% | 61.10% | 0.33 |
|  | High school or less | 48.40% | 38.90% |  |
| Maternal education level | Bachelor’s degree or above | 54.40% | 55.90% | 0.88 |
|  | High school or less | 45.60% | 44.10% |  |
| Paternal employment | Yes | 93.80% | 100.00% | 0.14 |
|  | No | 6.20% | 0.00% |  |
| Maternal  employment | Yes | 71.20% | 60.60% | 0.26 |
|  | No | 28.80% | 39.40% |  |
| Having past medical history | Yes | 56.50% | 50.00% | 0.48 |
|  | No | 43.50% | 50.00% |  |
| Having present illness | Yes | 26.10% | 27.50% | 0.86 |
|  | No | 73.90% | 72.50% |  |
| Outpatient visits | Mean(SD) | 0.83(1.93) | 0.70(1.39) | 0.66 |
| Absences | Mean(SD) | 0.42(1.64) | 0.15(0.70) | 0.16 |
| Lateness/  early leave | Mean(SD) | 0.10(0.53) | 0.13(0.46) | 0.76 |
| Infectious symptoms | Mean(SD) | 1.55(1.76) | 1.56(1.55) | 0.96 |
| EQ-5D | Mean(SD) | 0.97(0.06) | 0.95(0.08) | 0.21 |
| EQ-VAS | Mean(SD) | 86.70(12.56) | 84.97(14.39) | 0.50 |

| Control group | | | | |
| --- | --- | --- | --- | --- |
|  | | Complete the pre- and post-surveys  (n = 99) | Complete only the pre-survey  (n = 48) | p-value^1^ |
| Sex | Male | 47.50% | 47.90% | 0.96 |
|  | Female | 52.50% | 52.10% |  |
| Age | Mean(SD) | 10.37(1.74) | 10.77(1.67) | 0.19 |
|  | ≥10 years | 66.70% | 77.10% | 0.20 |
|  | <10 years | 33.30% | 22.90% |  |
| Having sibling(s) | Yes | 86.60% | 89.10% | 0.67 |
|  | No | 13.40% | 10.90% |  |
| Main caregiver | Mother | 71.70% | 75.00% | 0.67 |
|  | Others | 28.30% | 25.00% |  |
| Family type | Two parents | 70.70% | 62.50% | 0.32 |
|  | Others | 29.30% | 37.50% |  |
| Insurance type | NHI | 85.10% | 82.20% | 0.66 |
|  | Not NHI | 14.90% | 17.80% |  |
| Household income | ≥3000$ | 36.20% | 25.50% | 0.20 |
|  | <3000$ | 63.80% | 74.50% |  |
| Paternal education level | Bachelor’s degree or above | 56.30% | 67.50% | 0.23 |
|  | High school or less | 43.70% | 32.50% |  |
| Maternal education level | Bachelor’s degree or above | 40.30% | 69.00% | 0.00* |
|  | High school or less | 59.70% | 31.00% |  |
| Paternal employment | Yes | 95.30% | 94.90% | 0.91 |
|  | No | 4.70% | 5.10% |  |
| Maternal  employment | Yes | 68.30% | 70.70% | 0.78 |
|  | No | 31.70% | 29.30% |  |
| Having past medical history | Yes | 40.40% | 50.00% | 0.27 |
|  | No | 59.60% | 50.00% |  |
| Having present illness | Yes | 18.20% | 18.80% | 0.93 |
|  | No | 81.80% | 81.20% |  |
| Outpatient visits | Mean(SD) | 0.62(1.51) | 0.34(1.06) | 0.22 |
| Absences | Mean(SD) | 0.15(0.77) | 0.08(0.35) | 0.46 |
| Lateness/  early leave | Mean(SD) | 0.09(0.43) | 0.15(0.50) | 0.49 |
| Infectious symptoms | Mean(SD) | 1.09(1.62) | 1.00(1.62) | 0.75 |
| EQ-5D | Mean(SD) | 0.97(0.06) | 0.97(0.08) | 0.98 |
| EQ-VAS | Mean(SD) | 87.09(12.14) | 86.24(13.40) | 0.72 |

Abbreviations: NHI, National Health Insurance; SD, standard deviation

* p<0.05, statistically significant

^1^ This column presents *p-values* of chi-square tests for binomial variables and independent t-tests for continuous variables.
