# Supplementary material for: Evaluation of the effectiveness of the standard traditional Korean medicine-based health promotion program for disadvantaged children in South Korea
Source: BMC Complement Med Ther. 2022 Jun 26;22:175. doi: 10.1186/s12906-022-03634-w (PMC9233805; doi:10.1186/s12906-022-03634-w)
Supplement: Supplementary file 5 — Additional file 5. Goodness of fit test results. [file 12906_2022_3634_MOESM5_ESM.docx]

Additional file 5: Goodness of fit test results

| Overdispersion assumption: Poisson model vs NB model | | | | |
| --- | --- | --- | --- | --- |
| Likelihood-ratio test | | | | |
|  | Outpatient visits | Absence | Lateness /  early leave | Infectious symptoms |
| -2*Log likelihood  (Poisson model, df = 12) | -748.82 | -195.96 | -135.02 | -686.43 |
| -2*Log likelihood  (NB model, df = 13) | -460.71 | -137.16 | -118.43 | -645.85 |
| χ2 | 576.22 | 117.61 | 33.185 | 81.156 |
| p-value | 0.00* | 0.00* | 0.00* | 0.00* |
| Zero-inflated assumption: NB model vs ZINB model | | | | |
| Vuong test (AIC corrected) | | | | |
|  | Outpatient visits | Absence | Lateness /  early leave | Infectious symptoms |
| z-value | 3.59 | 2.076 | 0.592 | 1.432 |
| p-value | 0.00* | 0.02* | 0.277 | 0.076 |
| AIC | | | | |
|  | Outpatient visits | Absence | Lateness /  early leave | Infectious symptoms |
| NB model | 947.42 | 300.32 | 262.86 | 1317.70 |
| ZINB model | 921.21 | 278.42 | 258.06 | 1301.80 |

Abbreviations: df, degree of freedom; AIC, Akaike’s information criterion; NB, negative binomial; ZINB, zero-inflated negative binomial.

* p<0.05, Statistically significant
